# Supplementary material for: A low-cost hyperspectral scanner for natural imaging and the study of animal colour vision above and under water
Source: Sci Rep. 2019 Jul 25;9:10799. doi: 10.1038/s41598-019-47220-6 (PMC6658669; doi:10.1038/s41598-019-47220-6)
Supplement: Supplementary file 5 — Supplementary Information [file 41598_2019_47220_MOESM5_ESM.pdf]

Supplementary information for the manuscript

A low-cost hyperspectral scanner for natural imaging and the study of animal colour vision above and under water

N. E. Nevala[1] and T. Baden[1,2]

[1]School of Life Sciences, University of Sussex, United Kingdom

[2]Institute of Ophthalmic Research, University of Tübingen, Germany

$Correspondence at n.nevala@sussex.ac.uk and t.baden@sussex.ac.uk

Supplementary Video 1.

A video demonstrating the mirror movements and how light is guided to the spectrometer through them.

Supplementary Videos 2-4.

Hyperspectral reconstructions of the three scanned scenes presented in this work, with each frame corresponding to a 1 nm instance. Videos 2 and 3 in addition depict reconstructions of combined red, green and blue (RGB) channels for human spectral sensitivity of the scanned scene next to the videos. Video 4 instead depicts a combined zebrafish RGB reconstruction of the scene.
